# Non-invasive detection of a femoral-to-radial arterial pressure gradient in intensive care patients with vasoactive agents

**DOI:** 10.1186/s40560-021-00585-1

**Published:** 2021-11-27

**Authors:** Matthias Jacquet-Lagrèze, David Claveau, Julie Cousineau, Kun Peng Liu, Jean-Gilles Guimond, Pierre Aslanian, Yoan Lamarche, Martin Albert, Emmanuel Charbonney, Ali Hammoud, Loay Kontar, André Denault

**Affiliations:** 1 Centre Hospitalier Louis Pradel, Département d’Anesthésie Réanimation, Hospices Civils de Lyon, 59 Boulevard Pinel, 69500 Bron, France; 2 Université Claude-Bernard, Lyon 1, Campus Lyon Santé Est, 8 avenue Rockefeller, 69008 Lyon, France; 3 Carmen Laboratory, IHU OPERA, Inserm U1060, University Claude Bernard Lyon 1, Lyon, France; 4 Centre de Santé et de Svc, 435 rue Saint Roch, Trois-Rivières, QC G9A 2L9 Canada; 5 CHUM, 1051 rue Sanguinet, Montreal, QC H2X 3E4 Canada; 6 Pierre-Le Gardeur Hospital, 911 Montée des Pionniers, Terrebonne, QC J6V 2H2 Canada; 7 Montreal Heart Institute, Université de Montréal, 5000 rue Belanger, Montreal, QC H1T 1C8 Canada; 8 Hôpital du Sacré-Coeur de Montréal, 5400 boul. Gouin O, Montreal, QC H4J 1C5 Canada; 9 CHU Amiens-Picardie Site Nord, 2 Place Victor Pauchet, 80080 Amiens, France

**Keywords:** Diagnostic study, Aorto-radial gradient, Femoro-radial gradient, Vasoplegia

## Abstract

**Background:**

In patient requiring vasopressors, the radial artery pressure may underestimate the true central aortic pressure leading to unnecessary interventions. When using a femoral and a radial arterial line, this femoral-to-radial arterial pressure gradient (FR-APG) can be detected. Our main objective was to assess the accuracy of non-invasive blood pressure (NIBP) measures; specifically, measuring the gradient between the NIBP obtained at the brachial artery and the radial artery pressure and calculating the non-invasive brachial-to-radial arterial pressure gradient (NIBR-APG) to detect an FR-APG. The secondary objective was to assess the prevalence of the FR-APG in a targeted sample of critically ill patients.

**Methods:**

Adult patients in an intensive care unit requiring vasopressors and instrumented with a femoral and a radial artery line were selected. We recorded invasive radial and femoral arterial pressure, and brachial NIBP. Measurements were repeated each hour for 2 h. A significant FR-APG (our reference standard) was defined by either a mean arterial pressure (MAP) difference of more than 10 mmHg or a systolic arterial pressure (SAP) difference of more than 25 mmHg. The diagnostic accuracy of the NIBR-APG (our index test) to detect a significant FR-APG was estimated and the prevalence of an FR-APG was measured and correlated with the NIBR-APG.

**Results:**

Eighty-one patients aged 68 [IQR 58–75] years and an SAPS2 score of 35 (SD 7) were included from which 228 measurements were obtained. A significant FR-APG occurred in 15 patients with a prevalence of 18.5% [95%CI 10.8–28.7%]. Diabetes was significantly associated with a significant FR-APG. The use of a 11 mmHg difference in MAP between the NIBP at the brachial artery and the MAP of the radial artery led to a specificity of 92% [67; 100], a sensitivity of 100% [95%CI 83; 100] and an AUC ROC of 0.93 [95%CI 0.81–0.99] to detect a significant FR-APG. SAP and MAP FR-APG correlated with SAP (*r*^2^ = 0.36; *p* < 0.001) and MAP (*r*^2^ = 0.34; *p* < 0.001) NIBR-APG.

**Conclusion:**

NIBR-APG assessment can be used to detect a significant FR-APG which occur in one in every five critically ill patients requiring vasoactive agents.

## Key messages

A significant radial-to-femoral pressure gradient occurs in nearly one-fifth of patients in ICU. Repeated non-invasive assessment of the radial-to-brachial pressure gradient was significantly correlated with the invasive radio-femoral arterial pressure gradient.

## Background

Radial arterial pressure monitoring is a common practice in intensive care units (ICU) [1]. However, the radial artery blood pressure measurement can be inaccurate. In normal patients central-to-peripheral gradient with higher pressure in peripheral artery than in the aorta is typically observed [2]. This gradient typically affects the systolic component of the arterial pressure (SAP), while the mean arterial pressure (MAP) and diastolic arterial pressures (DAP) are usually unaltered. This peripheral-to-central gradient decreases with age [3]. During cardiopulmonary bypass [4–6] or during acute circulatory failure requiring high levels of vasopressors [7, 8], it has been reported that a significant abnormal central to peripheral gradient could occur, with higher pressure in the aorta compared to the peripheral artery. Unfortunately, in that case, all the components of the arterial pressure can be affected, and the clinician may underestimate the central arterial pressure, if one relies only on the radial artery pressure measurement.

Risk factors in detecting this abnormal arterial peripheral-to-central gradient have been identified during cardiac surgery, but the diagnostic accuracy of such an approach is low [9, 10]. This blood pressure (BP) gradient has significant clinical implication as it could lead the clinician to under evaluate the arterial BP leading to excessive fluid and/or vasopressor therapy. No test with enough accuracy [11, 12] can detect a significant central to peripheral arterial pressure gradient. Furthermore, the prevalence in the ICU of a significant peripheral to central arterial pressure gradient is rarely reported [7, 8, 13, 14]. As an alternative to radial invasive measurement, it has been reported that invasive and non-invasive blood pressure (NIBP) at brachial artery were higher [15, 16] and a better reflection of aortic measurement [17, 18]. Indeed, the pressure gradient between central arterial pressure and a point of measurement in the upper limb is proportional to the distance from the aorta to the point of measurement [19].

These observations raise the question about the possibility to detect such a gradient in clinical practice, and eventually identify the population which need an invasive assessment, beyond the sole radial measurement. Several studies have shown that femoral and aortic arterial pressure were equivalents [9, 17], but they have not been compared to NIBP measurements. The primary objective of the present study was to assess the accuracy of non-invasive assessment of the brachial-to-radial arterial pressure gradient (NIBR-APG) to detect the femoral-to-radial arterial pressure gradient (FR-APG) measured with invasive dual radial and femoral arterial BP monitoring. Secondary objectives were to correlate FR-APG and NIBR-APG and to evaluate the FR-APG prevalence in a sample of critically ill patients.

## Materials and methods

### Patients and settings

This prospective study was conducted in Montreal, Canada in three academic medical centers: over a 22-month period. The local Institutional Review Board for human subjects approved the study protocol (IRB#: MP-33-2016-1964) and informed consent was obtained before inclusion. We reported our study, regarding the methodological limits and the risk of bias, according to the STARD statement [20, 21]. Patients were eligible for inclusion if instrumented with a radial and a femoral arterial catheter and requiring a vasopressor to maintain a MAP of more than 65 mmHg. In one cardiac surgical institution, it was common practice to have both radial and femoral arterial monitoring, in the two other institutions the dual monitoring was at the discretion of the clinician in charge of the patient, mainly motivated by a suspicion of an FR-APG in patients facing high doses of vasopressors [22]. They were excluded in case of upper or lower limb amputation, arterial stenosis of the upper or lower limb, mechanical circulatory support, a moribund state, or when investigators were not available. We stopped all measurements (at T1, T2 or T3) once the vasoactive agents were weaned.

### Study protocol

The experimental protocol was as follows: when the patient was stabilized, we took exactly at the same time the two values of the radial and femoral arterial pressure (Philips M1006B Invasive BP Measurement Module InteliView MX; Philips, Amsterdam, Netherlands). This was collected right after the end of the NIBP done by the attending nurse using non-invasive oscillometric arterial pressure measured with an adapted cuff size at the brachial level on both sides. All the pressure measurements were recorded in semi-recumbent position within less than 10 min. Invasive arterial pressure was measured with an arterial catheter connected to a pressure transducer placed at the level of the phlebostatic axis (right atrium level: 4th intercostal along the mid axillary line), zero was set at the atmospheric pressure, a fast flush test was carried out before each measurement to check the harmonic characteristic of the system. Over-damping (less than 1 oscillation after the flush test) or underdamping (more than 2 oscillations after the flush test) were identified and corrected before measurement. The invasive and NIBP data were collected simultaneously three times with 1 h between each measurement at T1, T2, and T3. In most cases, T1 occurs in the first hours of the ICU stay. Notification of the invasive and non-invasive arterial pressure was not recorded blindly. At each timepoints, the heart rate, the ventilatory support variables, the doses of vasopressor and inotropes were recorded. The age, height, weight, reason for admission to the ICU and associated medical conditions were also collected. Lactate, creatinine, central venous oxygen saturation (ScVO_2_), cerebral near-infrared spectroscopy (NIRS), fluid balance at the time of the measurement if available and central body temperature values were noted. The Sequential Organ Failure Assessment Score [23] at inclusion and new Simplified Acute Physiology Score (SAPS2) [24] were collected. The FR-APG was defined as the difference between the invasive femoral and radial arterial pressure. The NIBR-APG was defined as the difference between the average of the NIBP recorded on the right and left upper limb and the radial arterial pressure. All the gradients were calculated based on both on the SAP and MAP.

### Endpoints and definitions

The primary objective was to evaluate the diagnostic accuracy of the NIBR-APG (our index test) to detect the FR-APG (our reference standard) in patients in ICU. Secondary objectives were to evaluate the correlation between the FR-APG and the NIBR-APG and to determine the prevalence of the FR-APG in our sample of patients. A significant BP gradient was defined as either an FR-APG of more than 25 mmHg using SAP or an FR-APG of more than 10 mmHg using MAP [9, 10].

### Statistical analysis

Sample size calculation was based on our primary objective. Using Obuchowsky’s method [25], 81 patients were needed to detect an area under curve (AUC) of the receiver operating characteristic (ROC) curve of 0.75 with a power of 0.8 and an alpha risk of 0.05. The ratio between patients with a significant FR-APG and patients without an FR-APG was hypothesized to be 0.15. The Shapiro–Wilk test determined the normal distribution of the data. For continuous data, the two-tailed Student *t* test or a Mann–Whitney *U* test to compare patients with and without a significant RCG. In the case of categorical data, we used a Chi^2^ test. Pearson’s correlation coefficient was used to test linear correlations. We built ROC curves and expressed AUC with 95% confidence interval (CI) calculated with a bootstrap method using 2000 repetitions. Sensitivity, specificity, positive and negative predictive values were expressed with 95%CI. We used the Free Software Foundation’s CRAN R to compute the statistical analysis. All tests were two-sided and a *p-*value < 0.05 was considered statistically significant.

## Results

**Fig. 1:**
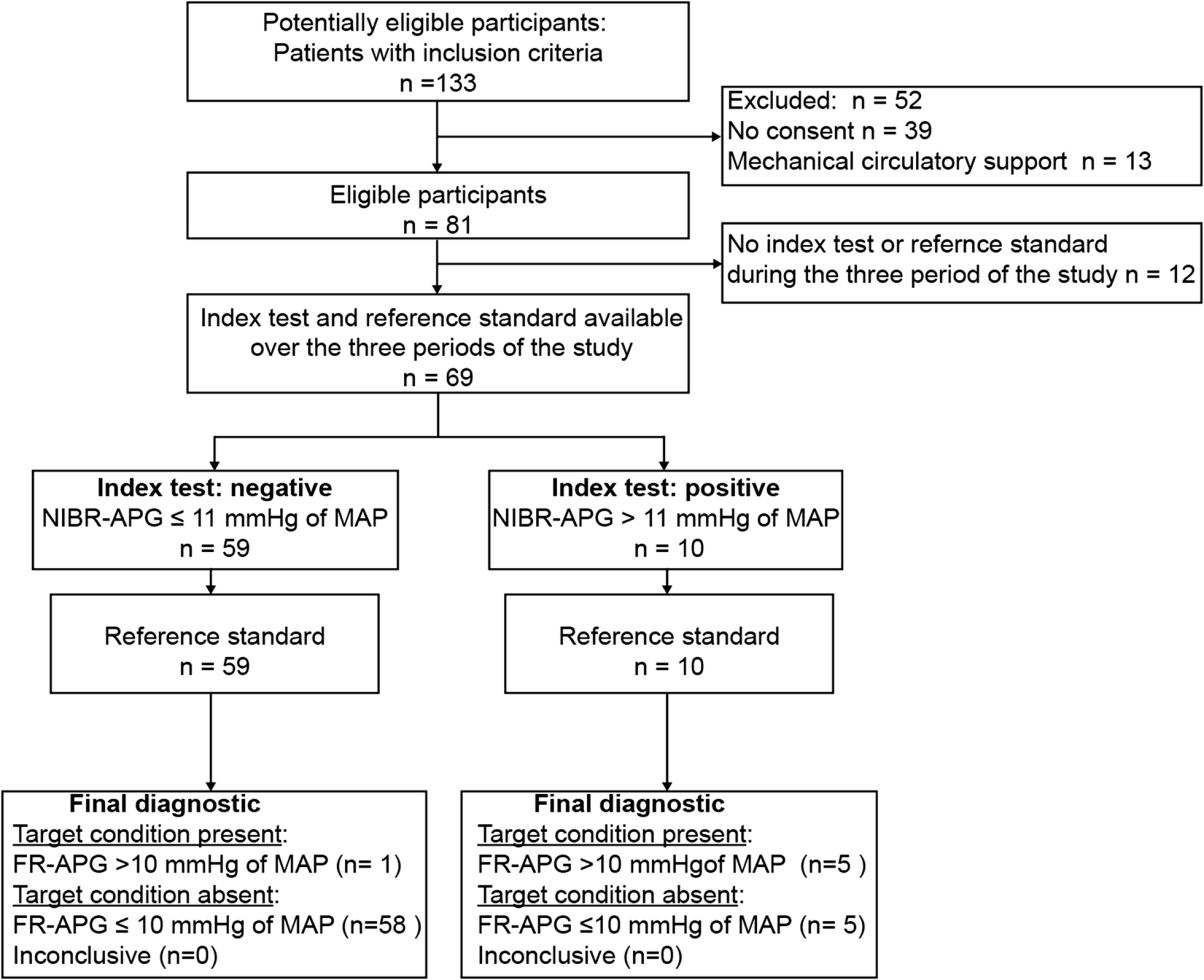
Flow chart. *FR-APG* femoral-to-radial arterial pressure gradient; *NIBR-APG* non-invasive brachial-to-radial arterial pressure gradient. The index test is the average NIBR-APG mean arterial pressure (MAP) measured three times (T1, T2, T3) with non-invasive cuff at the two arms at the brachial level and with an invasive radial artery cannulae. The “final diagnostic or reference standard” is defined as FR-APG of more than 10 mmHg of MAP measured with invasive femoral and radial cannulae

The flow chart of the study is reported in Fig. 1. A total of eighty-one patients were analyzed in which 228 measurements were obtained. The main demographic and clinical characteristics of all patients and those without and with a significant FR-APG are detailed in Table 1. Sixty-eight (84%) patients had benefited from cardiac surgery and thirteen were non-surgical cardiac patient mainly septic shock. We found 11 patients after cardiac surgery and 4 among non-cardiac surgery patients with a significant FR-APG. Patient with a significant FR-APG had significantly more diabetes (53% versus 23%, *p* = 0.044), higher lactate (2.8 [1.6, 5.2] mmol/L versus 1.8 [1.0, 2.4], *p* = 0.021) and higher dose of norepinephrine (0.30 [0.15, 0.50] µg/kg/min versus 0.15 [0.08, 0.25] *p* = 0.02). At T1, 81 measurements were available for the index test (NIBR-APG) and for the reference standard (FR-APG). At T2, 76 measurements of the reference standard and the index test were available (it was not measured for 5 patients who had no more norepinephrine infusion at that time). At T3, 71 measurements of the reference standard and the index test were available (it was not measured for 10 patients who had no more norepinephrine infusion at that time). The mean difference of the FR-APG using MAP was 4 mmHg at T1, ranging from − 6 to 28 mmHg; also 4 mmHg at T2 ranging from − 22 to 30 mmHg at T2 and 2 mmHg at T3, ranging from − 17 to 16 mmHg. The mean difference in the FR-APG using SAP was 10 mmHg at T1 ranging from − 14 to 62 mmHg; 8 mmHg at T2 ranging from − 17 to 58 mmHg and 2 mmHg at T3, ranging from − 17 to 16 mmHg.

**Table 1:** Patient’s demographic and clinical characteristics at T1

	All patients	No significant	Significant	*p* value
		FR-APG	FR-APG	
*n *(%)	81	66 (81)	15 (19)	
Gender, male/female, *n*	53/28	44/22	9/6	0.85
Age, year	68 [58, 75]	68 [58, 75]	70 [65, 76]	0.365
Height, cm	168 (10)	168 (10)	169 (11)	0.761
Weight, kg	80 [71, 94]	80 [71, 94]	81 [75, 87]	0.863
BMI, cm/kg^2^	29 (5)	29 (6)	29 (4)	0.848
Background				
Hypertension	53 (66%)	41 (63%)	12 (80%)	0.344
Diabetes melitus	23 (29%)	15 (23%)	8 (53%)	0.044
Ischemic cardiomyopathy	41 (51%)	36 (55%)	5 (33%)	0.231
Clinical context				
Cardiac surgery	68	57 (86%)	11(73%)	0.247
Septic shock	5	4 (6%)	1 (7%)	0.904
Other	8	5 (8%)	3 (20%)	0.162
Cardiac and metabolic function				
LVEF, %	54 [40, 60]	52 [40, 60]	56 [49, 65]	0.144
Lactate, mmol/L	1.95 [1.10, 2.70]	1.8 [1.0, 2.4]	2.8 [1.6, 5.2]	0.021
Temperature, °C	36.7 (0.7)	36.7 (0.7)	36.9 (1.0)	0.511
ScvO_2_, %	73 [68, 77]	73 [67, 77]	71 [70, 77]	0.76
Mean NIRS, %	69 [69, 70]	70 [69, 70]	69 [68, 69]	0.14
Fluid				
Fluid intake, mL	2472 [1833, 3189]	2475 [1805, 3275]	2315 [2113, 2600]	0.731
Fluid loss, mL	1362 [593, 2401]	1398 [635, 2637]	1325 [527, 1715]	0.507
Fluid balance, mL	1320 [612, 2014]	1300 [573, 1851]	1775 [934, 2619]	0.165
Creatinine, µmol/L	105 [81, 138]	103 [80, 127]	121 [89, 168]	0.379
Severity scores				
SAPS2	35 (7)	34 (7)	39 (10)	0.146
SOFA score	6 [4, 7]	5 [4, 7]	6 [5, 8]	0.218
Drugs				
Norepinephrine, µg/kg/min	0.16 [0.08, 0.28]	0.15 [0.08, 0.25]	0.30 [0.15, 0.50]	0.02
Epinephrine, µg/kg/min	0.07 [0.04, 0.10]	0.05 [0.04, 0.08]	0.09 [0.07, 0.10]	0.294
Vasopressine, ui/kg/min	2.40 [2.40, 2.40]	2.40 [2.40, 2.40]	2.40 [2.40, 2.40]	0.378
Dobutamine, µg/kg/min	3.00 [2.50, 5.00]	2.50 [2.50, 3.75]	4.00 [3.50, 4.50]	0.361
Milrinone, µg/kg/min	0.34 [0.25, 0.47]	0.31 [0.25, 0.41]	0.40 [0.35, 0.45]	0.411
Hemodynamic				
HR, beat/min	78 (12)	77 (12)	83 (11)	0.249
*Radial artery*				
SAP, mmHg	103 (17)	107 (16)	89 (13)	< 0.001
DAP, mmHg	54 (10)	56 (9)	46 (10)	< 0.001
MAP, mmHg	69 (11)	72 (9)	58 (10)	< 0.001
*Femoral artery*				
SAP, mmHg	113(15)	110 (13)	125 (18)	< 0.001
DAP, mmHg	55 (9)	56 (8)	50 (8)	0.009
MAP, mmHg	72 [68, 78]	72[68, 78]	70 [64, 77]	0.315

Variables are expressed as mean (standard deviation) or median [interquartile range] according to their distributions

*BMI* body mass index; *DAP* diastolic arterial pressure; *LVEF* left ventricular ejection fraction; *MAP* mean arterial pressure; *NIRS* near-infrared spectroscopy; *FR-APG* femoral-to-radial arterial pressure gradient; *HR* heart rate; *SAP* systolic arterial pressure; *SAPS2* Simplified Acute Physiology Score; *ScVO*_*2*_ central venous oxygenation saturation; *SIRS* systemic inflammatory response syndrome; *SOFA* Sequential Organ Failure Assessment

### Diagnosis accuracy of the NIBR-APG to detect an FR-APG

**Fig. 2:**
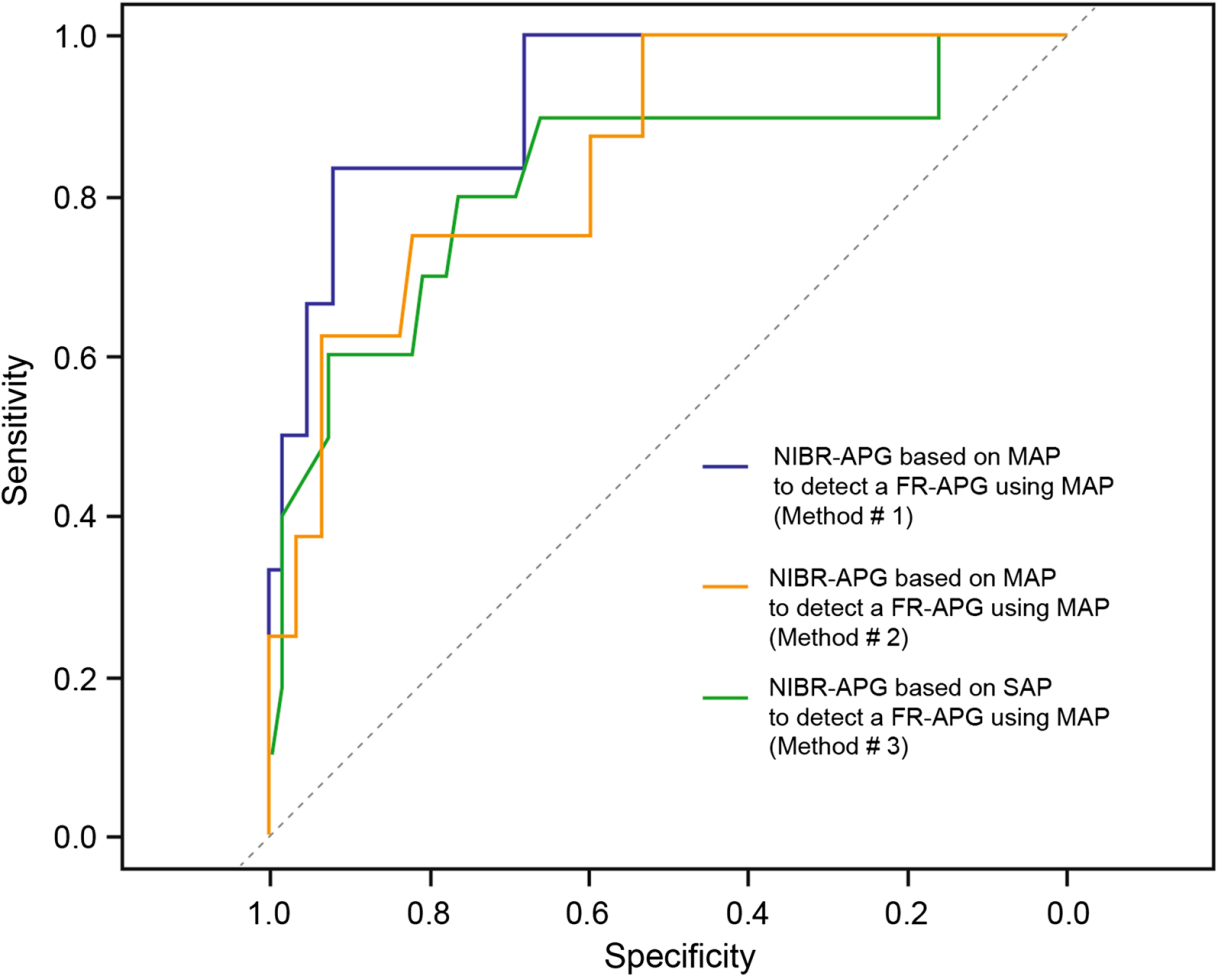
Receiver operating characteristics curves using three non-invasive methods to estimate the femoral-to-radial arterial pressure gradient (FR-APG) defined in terms of mean arterial pressure (MAP). The first method (purple) is using the average of the three non-invasive measurements (T1, T2 and T3) of the two upper limbs. The second method (orange) is using only non-invasive MAP value at T1. The third method (green) is using only non-invasive systolic arterial pressure (SAP) value at T1. *NIBR-APG* non-invasive brachial-to-radial arterial pressure gradient; *FR-APG* is defined as an FR-APG of more than 10 mmHg MAP measured with invasive femoral and radial cannulae

The diagnostic accuracy of the different components of the pressure gradient are detailed in Table 2 and Fig. 2. Among the six non-invasive approaches using the NIBR-APG to predict a significant FR-APG with either MAP or SAP, the most accurate evaluation was obtained using the average of the three measurements (T1, T2 and T3) of the two upper limbs using NIBP at the brachial artery level for the detection a significant FR-APG defined as a MAP difference of more than 10 mmHg. The use of a 11 mmHg difference in MAP between the NIBP at the brachial artery and the MAP of the radial artery led to a specificity of 92% [67; 100], a sensitivity of 100% [95%CI 83; 100] and an AUC ROC of 0.93 [95%CI 0.81–0.99].

**Table 2:** Diagnostic accuracy of non-invasive brachial-to-radial (NIBR-APG) arterial pressure gradient to detect a significant invasive mean and systolic femoral-to-radial arterial pressure gradient (FR-APG)

	Approaches	AUC–ROC [95%CI]	Best threshold (mm Hg)	Specificity	Sensitivity	PPV	NPV	Accuracy
MAP FR-APG (∆MAP > 10 mm Hg)	NIBR-APG avg (*n* = 66) (3 measurements)	0.93 [0.81–0.99]	11 [3; 16]	92 [67; 100]	100 [83;100]	50 [21; 100]	100 [97; 100]	91 [65; 99]
	NIBR-APG initial (*n* = 81) (1 measurement)	0.83 [0.61–0.95]	5 [-6; 11]	78 [62–97]	80 [60–100]	36 [23–75]	97 [94–100]	79 [64–95]
SAP FR-APG (∆SAP > 25 mm Hg)	NIBR-APG avg (*n* = 66) (3 measurements)	0.85 [0.71–0.97]	18 [-2; 27]	84 [53; 98]	88 [63; 100]	40 [20–83]	97 [94.100]	84 [53; 96]
	NIBR-APG initial (*n* = 81) (1 measurement)	0.80 [0.61–0.95]	14 [3; 25]	86 [66; 97]	75 [50; 100]	50 [28; 82]	95 [91; 98]	86 [70; 95]

*AUC-ROC* area under the curve of the receiver operating characteristic; *NPV* negative predictive value; *PPV* positive predictive value; *NIBR-APG avg* non-invasive brachial to radial arterial pressure gradient averaged from T1 to T3, considering the mean arterial pressure, at the radial level with a catheter and non-invasively on both arms with a cuff at the brachial level; *FR-APG* femoral-radio arterial pressure gradient, considering the mean arterial pressure, at the radial level with a catheter and with a femoral catheter to assess central arterial pressure; *NIBR-APG initial* non-invasive brachial to radial arterial pressure gradient at T1, considering the mean arterial pressure, at the radial level with a catheter and non-invasively on both arms with a cuff at the brachial level; *MAP* mean arterial pressure; *SAP* systolic arterial pressure gradient

### Correlation between NIBR-APG and FR-APG

**Fig. 3:**
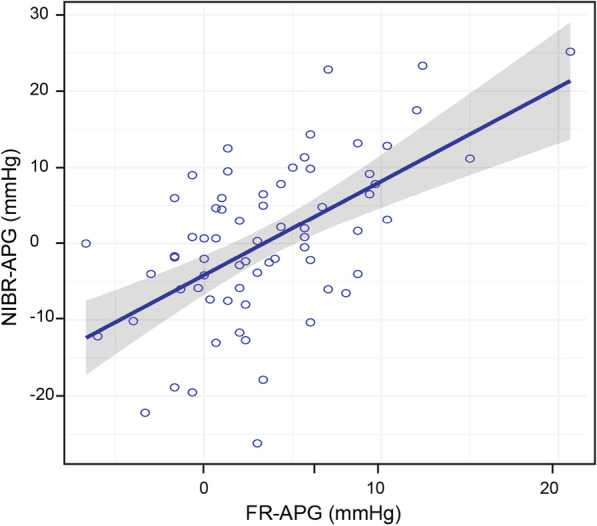
Scatter plot of the femoral-to-radial arterial pressure gradient (FR-APG) versus non-invasive brachial-to-radial arterial pressure gradient (NIBR-APG). The correlation between the FR-APG in mean arterial pressure (MAP) and the NIBR-APG in MAP was *r*^2^ = 0.32 (*p* < 0.001)

When comparing the MAP component of the FR-APG with the MAP of the NIBR-APG, the correlation was *r*^2^ = 0.34 (*p* < 0.001) at T1, *r*^2^ = 0.41 (*p* < 0.001) at T2, *r*^2^ = 0.21 (*p* < 0.001) at T3. When comparing the SAP component, the FR-APG with the SAP of the NIBR-APG, the correlation was *r*^2^ = 0.36 (*p* < 0.001) at T1, *r*^2^ = 0.41 (*p* < 0.001) at T2, *r*^2^ = 0.35 (*p* < 0.001) at T3. When averaging the measurements over time (T1 to T3), the SAP component (*r*^2^ = 0.30, *p* < 0.001) and the MAP component (*r*^2^ = 0.32, *p* < 0.001) of the FR-APG and the NIBR-APG were significantly correlated (Fig. 3).

### Prevalence of the FR-APG

Overall, at T1, 15 patients, 18.5% [95%CI 11–29%], at T2, 18 patients, 24% [95%CI 15–35] and at T3, 8 patients 12.5% [95%CI 15–35] had a significant SAP or MAP FR-APG. In terms of SAP and MAP gradient values, at T1, 12 patients (15%) [95%CI 8–24] had a significant SAP FR-APG and 10 (12%) [95%CI 6–22] had a significant MAP FR-APG. At T2, 10 patients (13%) [95%CI 7–23] had both a significant SAP FR-APG and MAP FR-APG. At T3, 7 patients 10% [95%CI 7–24] had a significant SAP FR-APG and 3 patients (4%) [95%CI 1–12] had a significant MAP FR-APG.

## Discussion

The main finding of our study is that 15 patients among 81 patients (18.5% [95%CI 11–29%]) had a significant FR-APG in the ICU. Patients with abnormal FR-APG were more frequently diabetes, with higher lactate and received higher doses of norepinephrine. We also found that the average measurements of the two arms every hour, three times, of the NIBR-APG was significantly correlated with the FR-APG. Such measurement can diagnose a significant FR-APG with an AUC ROC of 0.93 [95%CI 0.81–0.99]. The best threshold was a 11 mmHg difference between the MAP of the brachial NIBP and the MAP of the radial artery pressure, with a specificity of 92% [67; 100] and a sensitivity of 100% [95%CI 83; 100].

The prevalence of a significant gradient on our selected group of patients was lower than what has been described during cardiac surgery, but is concordant with another similar ICU study [7]. In Kim study, 37 septic shock patients were monitored with both radial and femoral arterial pressure [7]. Higher FR-APG (using an MAP) > 10 mmHg were observed in 27% of patients. Femoral pressures were found to be higher than radial particularly in the group receiving high dose of noradrenaline (> 0.1 μg/kg/min) as reported by Dorman et al. [8]. The abnormal FR-APG does not seem to be a rare event in ICU and can certainly affect the therapeutic decision with the risk of excess of fluid [26–31] and vasopressors [32–38] which both have been reported to be harmful.

There is a discrepancy in the different studies in the identification of risk factors of a significant gradient. As previously described in the ICU, significant gradient is associated with higher doses of vasopressors [7, 22, 39, 40]. The association with higher norepinephrine dosage is thought to be part of the physiological mechanism underlying this condition which is consistent with our findings [41, 42]. In fact, vasoactive agents are known to create peripheral vasoconstriction of medium-size arteries [12, 42] which may also explain the higher lactate that we observed.

Kanazawa et al. studied different site of measurement along the radial, brachial and the subclavian artery. They found a strong correlation between the distance from the aorta to the site of measurement and the pressure drop from the aorta to the site of measurement [19]. Those findings are consistent with the fact that the non-invasive assessment of the brachial pressure enables the calculation of a gradient with the radial artery that was correlated with the FR-APG. The detection of the MAP gradient seems more accurate than the SAP gradient this could be explained by the reduced accuracy of the oscillometric method to assess SAP compared with MAP [1].

This study has several limitations. First, the association of FR-APG with diabetes has not been reported previously but our number of patients with a significant FR-APG is too small to identify more than one factor using logistic regression. This is, however, the largest reported study with radial and femoral artery measurements in the ICU after Mignini who included 55 patients [13] and the first reporting NIBP to estimate the FR-APG. Second, most of the patients are from a cardiac surgical intensive care unit despite the fact that there were three other medico-surgical ICU involved. This can be explained by the more frequent use of dual radial and femoral artery pressure monitoring in that population. This selection bias could affect the prevalence of the gradient as cardiopulmonary bypass have been described as a significant risk factor for FR-APG [9, 10]. The precise duration of the FR-APG was not evaluated, but we observed a reduction in the FR-APG over time resulting from reduction in vasoactive agents based on the femoral arterial pressure values. However, the reduction in the severity of the gradient was influenced by an unblinded use of the femoral artery as a target to wean vasoactive agents. In the absence of a femoral artery and detection of an FR-APG, the duration of vasoactive support could be longer. A recent study from our center has shown that the use of combined radial and femoral artery catheter versus radial artery alone was associated with a reduction in the duration of vasoactive agents used in the ICU despite using dual radial and femoral arterial pressure in sicker patients [43]. Third, the precision of the NIBP assessment was superior when averaged on three measurements as when only one measurement was made. This is similar to performing more than one thermodilution cardiac output to increase the precision of the measurement. Fourth, there is a risk of biases as the nurse in charge who performed all the measurements was not blind from the reference standard (invasive femoral and radial arterial pressure) when collecting the data of the index test and vice versa. Nevertheless, it is unlikely that the absence of blindness affected the results significantly. Finally, FR-APG can still be present in the absence of vasoactive agents. In our study, we stopped the protocol when those agents were weaned which could led to underestimation of the FR-APG prevalence.

There are numerous advantages of continuous invasive arterial pressure measurement even through the radial artery which include blood gases measurements. If a discrepancy occurs and persist between non-invasive measurements and the radial artery, inserting a femoral arterial line would be suggested. Finally, an indication bias might be present. The use of both radial and femoral arterial pressure monitoring might have been decided for patients at risk of developing such gradients. However, in our institution, the use of both monitoring site is routinely used in up to 70% of patients in cardiac surgical patients [9, 43].

## Conclusion

Significant FR-APG can occur in nearly 1 out of 5 ICU patients. Such can affect significantly the interpretation of the hemodynamic condition of our patient with a risk of fluid or vasopressors overuse. No clear risk factors were linked strongly enough to enable the clinician to rule out the presence of that condition on an epidemiological approach. The NIBP measurement of brachial arterial using a MAP threshold of 11 mmHg can identify those patients and may avoid the use of an invasive femoral catheter. Persistence of a gradient despite modification of therapeutic strategy could warrant more invasive diagnostic strategy. Validation of this hypothesis in a larger cohort of patients should be performed.

## Data Availability

Not applicable.

## Abbreviations

AUC: Area under the curve
BP: Blood pressure
CI: Confidence interval
DAP: Diastolic arterial pressure
FR-APG: Femoral-to-radial arterial pressure gradient
ICU: Intensive care unit
MAP: Mean arterial pressure
NIBP: Non-invasive blood pressure
NIBR-APG: Non-invasive brachial-to-radial arterial pressure gradient
NIRS: Near infrared spectroscopy
ROC: Receiver operating curve
SAP: Systolic arterial pressure gradient
SAPS2: Simplified Acute Physiology Score II
ScVO_2_: Central venous oxygen saturation
